# The Road to Cancer Care: Understanding How Far Owners Travel for Their Pets’ Oncology Treatment

**DOI:** 10.3390/vetsci13010034

**Published:** 2025-12-31

**Authors:** Angus Lane, Kelly L. Bowlt Blacklock, Laura Blackwood

**Affiliations:** Royal (Dick) School of Veterinary Studies, University of Edinburgh, Easter Bush Campus, Roslin EH25 9RG, UK; kelly.blacklock@ed.ac.uk (K.L.B.B.); laura.blackwood@ed.ac.uk (L.B.)

**Keywords:** oncology, referral, travel, dog, cat, insurance

## Abstract

Access to specialist veterinary oncology services may be influenced by geographic, demographic, and patient-related factors. Understanding travel burden is important for identifying potential barriers to care and designing more equitable service delivery models. This study quantified the distance travelled by owners seeking specialist oncology care at a UK veterinary teaching hospital and examined whether species, age, breed, and insurance status were associated with travel patterns. A retrospective review was conducted of all dogs and cats presenting to the Oncology Service at the Hospital for Small Animals, University of Edinburgh, between 1 December 2018 and 31 October 2025. Owner postcodes were used to calculate distances from residence to the hospital. Distances were compared across species (dog vs. cat), breed (pure-breed vs. mixed-breed), age (<7 vs. ≥7 years), and insurance status (insured vs. uninsured). A total of 3074 cases were included. In univariate analysis, dogs travelled significantly further than cats (*p* < 0.001), pure-breed animals travelled significantly further than mixed-breed animals (*p* < 0.001), and younger animals travelled significantly further than older animals (*p* = 0.002). In multivariate analysis, species, age, and insurance status were significant, with dogs (*p* < 0.001), younger animals (*p* = 0.012), and uninsured animals (*p* = 0.008) travelling further. These findings highlight potential geographic inequities in access to specialist care and underscore the need for alternative service-delivery strategies to improve accessibility, particularly for cats and younger animals.

## Simple Summary

This study explores whether some pets face greater challenges than others in reaching specialist cancer care, focusing on the distance owners must travel. Long travel distances can make treatment harder to access and may prevent some pets from receiving specialist care. The aim of this work is to measure how far owners of dogs and cats travel to a specialist veterinary cancer service in the United Kingdom, and to understand whether factors such as the pet’s species, age, breed, and insurance status influence travel patterns. We reviewed records from over three thousand dogs and cats seen at a university veterinary teaching hospital over several years, and calculated the distance from each owner’s home to the hospital. We found that owners of dogs travelled further than owners of cats, younger animals travelled further than older animals, and animals without insurance travelled further than insured animals. These findings suggest that access to specialist veterinary cancer care is not equal for all pets. Recognising these differences can help veterinary services develop new ways of delivering care that are more accessible, improving fairness and outcomes for animals and their owners in society.

## 1. Introduction

Access to specialist veterinary oncology services is increasingly recognised as an important component of companion animal cancer care [1,2]. As the number of pets diagnosed with neoplastic disease continues to rise [3,4], so too does demand for advanced diagnostic and therapeutic options delivered by board-certified oncologists [5,6,7]. However, specialist oncology centres in the United Kingdom are comparatively few in number and geographically unevenly distributed, leading many owners to travel substantial distances to access care [8]. Travel burden is an important yet understudied determinant of access to veterinary oncology services, with potential implications for equity, timely diagnosis, treatment decisions, and ultimately patient outcomes.

A small number of studies have explored the broader factors that influence owner decision-making in veterinary cancer care, including pet insurance status, financial resources, comorbidities, and perceived quality of life. For example, owners of insured dogs are more likely to pursue advanced diagnostics and treatment options [9,10], including chemotherapy for lymphoma, while cats face greater barriers to veterinary care due to transport stress and owner perceptions of travel difficulty [11,12,13,14,15,16]. Despite the recognised influence of these factors on treatment uptake, the relevance of geographic accessibility—defined as the ease with which owners can physically reach a specialist centre—remains poorly quantified in veterinary medicine.

In human oncology, the impact of travel distance on access to care has been extensively documented [17,18,19,20,21]. Patients living farther from specialist centres often experience delays in diagnosis, lower likelihood of curative-intent treatment, and in some cases, poorer outcomes [22]. Younger patients—such as children and adolescents with cancer—frequently travel long distances (sometimes exceeding 60 miles per round trip) to access tertiary treatment facilities [23], whereas older adults may be less willing or able to undertake long journeys, even when high-volume specialist hospitals provide superior outcomes [24]. These human healthcare patterns closely parallel the authors’ experiences in veterinary oncology and highlight the potential importance of travel burden as a determinant of treatment access in companion animals.

The aim of this study was to quantify the distances travelled by owners seeking specialist oncology care at a UK veterinary teaching hospital and to examine whether species, age, breed, and insurance status influence travel patterns. By characterising geographic accessibility in a large cohort of patients, this work aims to provide foundational data to support future service planning, inform the development of alternative models of oncology care delivery such as tele-oncology [25], and enable meaningful comparison with established patterns in human healthcare accessibility research [22].

## 2. Materials and Methods

This study received ethical approval from the Human Ethical Review Committee of the Royal (Dick) School of Veterinary Studies, University of Edinburgh (protocol code HERC_2025_159), and the Veterinary Ethical Review Committee of the Royal (Dick) School of Veterinary Studies, University of Edinburgh (protocol code 233.25).

Patient and owner data were obtained for all canine and feline cases presenting to the Oncology Service at the Hospital for Small Animals, University of Edinburgh, from 1 December 2018 to 31 October 2025. Patient species, age at first presentation, breed, and insurance status were recorded, as well as the address of the owner. From this information, the coordinates (expressed as latitude and longitude) of each owner’s residence were calculated. The distance from the coordinates of their residence to the Hospital site was calculated using the Haversine formula, a method of calculating great-circle distances between two points on a sphere. This measures crow-flies distance, rather than actual road distance; previous research has shown this method to give acceptable levels of accuracy [26]. Patients were excluded if owner’s residence, species, breed, or age data were incomplete or absent. Descriptive statistics were generated for all variables. Distributional assessment was performed using histograms, Q-Q plots, and the Shapiro–Wilk test. Distance travelled showed a markedly right-skewed distribution and did not meet assumptions of normality (Shapiro–Wilk *p* < 0.001), and therefore summary statistics are presented as medians with interquartile ranges, and multivariable analyses were conducted using a log-transformed distance variable to improve normality of residuals and stabilise variance. Categorical data was presented as frequencies and percentages. A freely available webtool was used to depict median distances travelled [27], which were then exported to Google Earth Pro version 7.3.6 (Google LLC, Mountain View, CA, USA) [28].

Insurance status was analysed as a binary variable (insured vs. uninsured). Univariable associations between insurance status and species, breed group (pure-breed vs. mixed-breed), and age category (<7 years vs. ≥7 years) were assessed using Pearson’s chi-squared tests. All variables were then entered into a multivariable binary logistic regression model to identify independent predictors of insurance status, with results reported as odds ratios with 95% confidence intervals. Model fit was assessed using the Hosmer–Lemeshow test, and collinearity was evaluated using Variance Inflation Factors.

Univariable comparisons of distance travelled between groups (species: dog vs. cat; breed group: pure-breed vs. mixed-breed; age group: <7 years vs. ≥7 years; insurance status: insured vs. uninsured) were carried out. Additional analysis was performed within the mixed-breed group, where the distance travelled for “designer” mixed-breeds were compared to traditional mixed-breeds. “Designer” mixed-breeds were defined as named cross-breeds, for example, cavapoo, cavachon, cockapoo, etc. All univariable analyses were performed using the Mann–Whitney U test. All variables were evaluated in multivariable analysis. To account for the right-skewed distribution of distance travelled, a multivariable linear regression model was constructed using a log-transformed distance as the dependent variable. Species, breed group, age group, and insurance status were entered as independent variables. Model assumptions, including homoscedasticity and normality of residuals, were evaluated using standard diagnostic plots. All statistical analyses were performed using IBM SPSS Statistics version 29 (IBM Corp., Armonk, NY, USA). Statistical significance was set at *p* < 0.05 for all analyses.

## 3. Results

Three thousand one hundred and thirty-nine (3139) patients were identified. Three non-canine or feline patients (one African Grey Parrot and two ferrets) were excluded. Sixty-two patients were excluded due to absent or incomplete data. Thus, 3074 patients were included in the study (Table 1). Of these 3074 patients, 2642 (85.9%) were dogs and 432 (14.1%) were cats. Overall, 2354 (76.6%) were pure-breed, while 720 (23.4%) were mixed-breed, and 2329 (75.8%) were aged 7 years or more, while 745 (24.2%) were aged under 7 years.

Of the 2642 dogs, 2240 (84.8%) were pure-breed, while 402 (15.2%) were mixed-breed, 113 (28.1%) of which were considered “designer” mixed-breeds. Of the 432 cats, 114 (26.4%) were pure-breed, while 318 (73.6%) were mixed-breed.

Of the 2642 total dogs, 1950 (73.8%) were insured, 1649 (84.6%) of which were pure-breed. Six hundred and ninety-two (692) dogs (26.2%) were uninsured, 591 (85.4%) of which were pure-breed. Of the 432 cats, 300 (69.4%) were insured, while 132 (30.6%) were not insured. Of the 114 pure-breed cats, 80 (70.2%) were insured, while of the 318 mixed-breed cats, 220 (69.2%) were insured. Among the 2329 older animals (≥7 years), 1671 (71.7%) were insured and 658 (28.3%) were uninsured, while among the 745 younger animals (<7 years), 579 (77.7%) were insured and 166 (22.3%) were uninsured.

### 3.1. Younger Animals Are Significantly More Likely to Be Insured

Univariable chi-squared analysis showed that insurance status did not differ significantly between species (*p* = 0.058) or between pure-breed and mixed-breed animals (*p* = 0.564). Age category, however, was significantly associated with insurance status (*p* = 0.001), with younger animals (<7 years) more likely to be insured than older animals. In the subsequent multivariable binary logistic regression model (including species, breed group, and age group), age group remained a significant predictor of insurance status (OR 0.734, 95% CI 0.604–0.892, *p* = 0.001), indicating reduced likelihood of insurance among older animals. Species and breed group were not statistically significant after adjustment (*p* = 0.058 and *p* = 0.564, respectively). The model demonstrated acceptable fit (Hosmer–Lemeshow *p* = 0.418), and collinearity was minimal (all VIF values 1.004–1.301).

### 3.2. Dogs, Pure-Breed Animals, Younger Animals, and Uninsured Animals Are More Likely to Travel Further to Access Oncological Care

**Figure 1:**
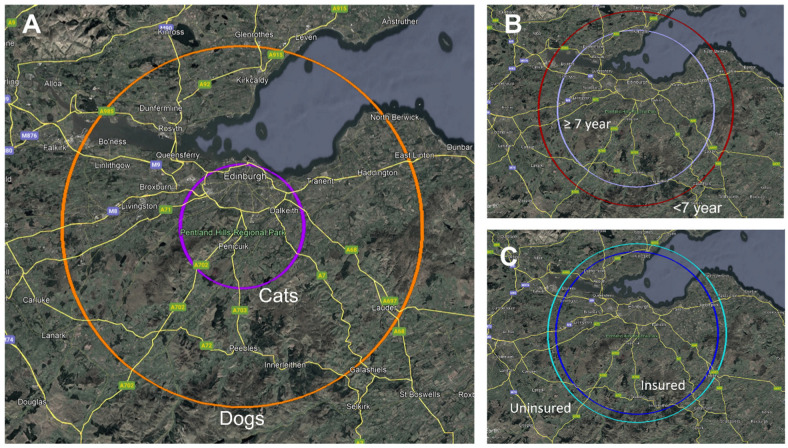
Circles of different radii centred on the Hospital for Small Animals, University of Edinburgh, used to depict median distance travelled between groups. (**A**) shows the median distance travelled for dogs (34 km, orange) and cats (12 km, violet). (**B**) shows the median distance travelled for younger animals (<7 years, 36 km, burgundy) and older animals (≥7 years, 29 km, lilac). (**C**) shows the median distance travelled for uninsured (33 km, aqua) and insured (30 km, royal blue) animals.

The median distance travelled for all patients was 30 km (IQR 52 km). The median distance travelled, quartiles, and interquartile range for different groups are outlined in Table 1. The median distance travelled for dogs, cats, pure-breed patients, and mixed-breed patients are illustrated in Figure 1.

In univariable analysis, dogs, pure-breed animals, and younger animals (<7 years) were significantly more likely to travel greater distances to access oncological care (Table 1). Insurance status was not statistically significant (*p* = 0.078). There was no significant difference in distance travelled between “designer” mixed-breed dogs when compared to “standard” mixed-breed dogs (*p* = 0.173). Species, age category, and insurance status were all significant predictors.

In the multivariable linear regression model assessing how the categorical factors species, breed, age category, and insurance status were associated with log-transformed distance travelled, the overall model was statistically significant (*p* = 0.001), but explained a small proportion of the variance (R^2^ = 0.026). Species, age category, and insurance status were all significant. Dogs travelled significantly further than cats (B = 0.47, 95% CI 0.339–0.600; Exp(B) = 1.60, 95% CI 1.40–1.82; *p* < 0.001). Conversely, older animals (≥7 years) travelled shorter distances than younger ones (B = −0.119, 95% CI −0.213 to −0.026; Exp(B) = 0.89, 95% CI 0.81–0.97; *p* = 0.012). Insured animals also travelled shorter distances compared with uninsured animals (B = −0.122, 95% CI −0.213 to −0.032; Exp(B) = 0.89, 95% CI 0.81–0.97; *p* = 0.008). Collinearity diagnostics showed no evidence of problematic multicollinearity among the predictors, with VIF values ranging from 1.004 to 1.301.

## 4. Discussion

This study aimed to evaluate whether species, breed, age, and insurance status influence the distance owners travel to access specialist veterinary oncology care. The results demonstrate that species, age, and insurance status are the primary factors associated with distance travelled by patients presenting to the Hospital for Small Animals Oncology Service. In univariate analysis, dogs travelled significantly further than cats, pure-breed animals travelled further than mixed-breeds, and younger animals (<7 years) travelled further than older animals (≥7 years), with insurance status approaching but not meeting statistical significance. When these variables were assessed together in a multivariable linear regression model, species and age remained independent predictors of distance travelled, with dogs travelling significantly further than cats, and younger animals travelling further than older animals, after adjusting for other variables. Interestingly, insurance status was not significant in univariable analysis but became significant after adjustment for species, breed, and age. This suggests a suppression effect, whereby confounding between insurance and other variables obscured the association when examined in isolation. Breed did not contribute additional explanatory value in the adjusted model. In spite of these findings, the overall variance explained by the model was low. These findings broadly align with established trends in veterinary healthcare utilisation and contribute insight into the geographic and demographic factors shaping access to veterinary oncology services.

The greater travel distance observed in the dog cohort compared with cats likely reflects several owner- and patient-related factors. Engaging with specialist care may be affected by financial factors. While older data suggests dog owners spend more on veterinary fees, recent reports suggest that the lifetime spend is similar [29,30,31,32]. A reluctance to undertake advanced diagnostic or therapeutic options may therefore be influenced by proximity of care, rather than simple financial considerations [33,34]. Conversely, dogs are more commonly insured than cats, with a 2024 survey of UK pet owners showing higher uptake in dogs (64%) than in cats (often 45%) [35]. A desire for access to specialist treatments may motivate owners to insure their pets, while availability of insurance funds may allow owners to undertake specialist treatment, which may require travel. However, owners may perceive dogs as more behaviourally adaptable to travel, whereas cats often exhibit heightened transport stress, which could reduce owner willingness to pursue long-distance specialist care [11,36]. Previous studies have reported that cats face greater barriers to veterinary care than dogs, partly due to owner perceptions of stress associated with transport, but also due to the stress of clinic visits [12,13]. Interestingly, dog ownership has been strongly associated with greater car distance travelled per person, even after accounting for income and urbanisation, suggesting dog owners are likely to be ready and able to travel [34].

The authors have observed that cat owners attending their clinic are more likely to travel via public transport and not have access to cars, but data regarding comparisons of car ownership in cat and dog owners is lacking [33,37,38]. In spite of the Hospital for Small Animals being well serviced by bus routes, this may serve as an additional barrier to referral for cat owners [39]. The present results suggest that multiple barriers may influence cat owners’ willingness to travel long distances for specialist oncology services. Provision of carrier training to cats has previously been shown to improve Cat Stress Score for car journeys to veterinary clinics, and implementation of this more widely may aid owners reluctant to travel further due to fears of impact on cat stress levels [38].

In the present study, age was significantly associated with travel distance in both univariate and multivariable analysis, suggesting that age meaningfully influences referral travel patterns among owners seeking veterinary oncology care. This finding agrees with expectations based on existing clinical reasoning, as age frequently factors into therapeutic decision-making for both veterinarians and owners [40]. Older animals with cancer often have comorbidities that may limit suitability for curative-intent therapies, and quality-of-life considerations commonly lead to more conservative treatment preferences in geriatric patients—such factors could theoretically reduce motivation for long-distance referral. This is reflected in the human literature, where a study investigating human patients with rectal cancer showed that elderly patients are less likely to travel long distances, even when high-volume specialist centres are further away [24]. Owners of younger animals—perceived as having greater potential treatment benefit or longer anticipated lifespan or healthspan—might be expected to be more inclined to travel further for specialist care.

Although pure-breed animals travelled farther than mixed-breed pets in univariate analysis, this association did not persist in the multivariable model, suggesting that breed itself is unlikely to be an independent driver of travel distance. Pure-breed and pedigree animals are often linked with higher expected healthcare costs and, in some populations, slightly increased insurance uptake, which might be expected to support greater willingness among owners to pursue specialist referral and therefore travel further for oncology care [41]. In addition, breed-associated predispositions to specific cancers—particularly in well-recognised high-risk breeds such as Boxers, Golden Retrievers, and Bernese Mountain Dogs—may also heighten owner vigilance and encourage earlier or more proactive referral behaviours [3,42]. However, it is possible that these factors may be counterbalanced by emerging trends in ownership of “designer” mixed-breeds, many of which demonstrate high insurance uptake and substantial owner investment comparable to or exceeding that seen in some pure-breed groups [43,44]. The mixture of potentially opposing socioeconomic and behavioural influences across pure-breed and mixed-breed populations may therefore dilute any true association, contributing to the absence of a significant breed effect in multivariable analysis.

Insurance coverage has been shown to increase acceptance of more expensive advanced diagnostics and referral-level treatment, including that of multicentric lymphoma in [9,10]. The finding that uninsured animals travelled farther than insured animals in the multivariable model was unexpected, as intuitively, more insured animals would be expected to travel for advanced treatment. One might reasonably expect insured owners, who face fewer direct financial barriers to specialist care, to be more willing or able to travel longer distances for oncology referral. However, the results in this study likely reflect a more complex interaction between socioeconomic factors, geographic location, and referral behaviour that is not fully captured in the dataset.

One plausible explanation is that uninsured owners may be more geographically dispersed than insured owners, for example, those in rural settings compared to urban settings, leading to farther distances travelled for referral care. It has previously been described that the insurance rate of dogs living in London (an urban area) was 39.6%, compared to those in Wales (a mostly rural to semi-rural area) of 16.4% [45]. Younger animals (<7 years) were more likely to be insured than older animals in both univariable and multivariable analysis. This pattern aligns with broader trends reported in companion animal health data, where insurance uptake is typically highest in younger pets and declines with age [45,46]. This may reflect increasing insurance premiums, policy restrictions, and reduced owner willingness to initiate or maintain coverage in older animals [46].

Socioeconomic constraints may further influence this pattern. Insurance status is an imperfect surrogate for financial capacity and does not capture broader indicators such as household income, employment flexibility, or access to transportation. Unlike insured owners, who may perceive the overall financial burden as partially mitigated by coverage of diagnostic and treatment costs, uninsured owners must typically absorb both direct medical fees and these ancillary expenses in full. As a result, uninsured owners who ultimately proceed with referral may represent a highly selected subgroup with sufficient financial resilience, flexible employment, reliable transportation, or strong motivation to pursue specialist care despite these barriers. Conversely, some insured owners may have sought insurance to reduce concerns regarding cost barriers, but lack the flexibility to access all available care. This selection effect may bias the observed travel distances upward for uninsured animals. Ultimately, the finding that uninsured animals travelled further should not be interpreted as evidence that lack of insurance facilitates access to referral care. Rather, it likely reflects underlying geographic and socioeconomic disparities that have not been captured in this dataset, which should be aimed to be assessed in future studies.

While the study offers valuable insights, several limitations must be acknowledged. Travel distance was calculated using straight-line (Haversine) distance rather than road network distance. This will underestimate the true relative travel burden experienced by owners in some settings. This limitation is likely to be most pronounced in regions with poor road infrastructure, indirect or fragmented transport networks, or limited availability of private vehicle access. In such settings, actual travel time and effort may substantially exceed that implied by linear distance alone—particularly for owners reliant on public transportation, multiple transport modes, or infrequent rural services. Regardless, straight-line measurement has been widely used in healthcare access research due to its consistency and objectivity; it also allows for standardised comparisons across large geographic areas and heterogenous populations [26]. The retrospective design limits the ability to capture additional influencing factors such as household income and transport availability. These variables have documented impacts on veterinary healthcare decisions and may help explain some of the variation observed [47,48].

The multivariable model explained only a small proportion of the overall variance in travel distance (R^2^ = 0.026), indicating that the predictors included—species, breed type, age, and insurance status—account for only a limited amount of the observed variability. Although statistically significant, largely due to the large sample size, this low R^2^ suggests that factors not captured within the dataset are likely to play a significant role in influencing how far clients travel for oncology referral. Socioeconomic variables are likely to be particularly important. Household income, car ownership, employment flexibility, and access to paid leave may all affect an owner’s ability or willingness to travel longer distances for specialist care. Insurance status may act only as a crude proxy for financial capacity and does not capture out-of-pocket costs such as fuel, accommodation, or time away from work—which may represent significant barriers for some insured clients. Similarly, access to reliable transportation and public transit infrastructure is likely to vary geographically and may disproportionately affect owners living in rural/remote areas or those with lower household incomes. Clinical factors not included in the model may also meaningfully influence travel decisions. The type of cancer, perceived prognosis, availability of treatment options, and urgency of referral are all likely to affect owner motivation to travel. Referring veterinarian recommendations, including the strength of the endorsement for specialist care or familiarity with specific oncology centres, may further shape client decision-making. Owner attitudes, expectations, and prior experiences with referral care are additional unmeasured behavioural factors that may contribute to travel patterns.

Despite these limitations, the results underscore important disparities in access to specialist oncology services, especially between cats and dogs, younger and older animals, and animals of different insurance status. Recognising these patterns is crucial for designing more equitable and scalable models of oncology service delivery. Tele-oncology services represent one promising solution, enabling remote consultations, follow-ups, and decision support for general practitioners, thereby reducing the need for long-distance travel [25]. The development of regional satellite clinics or collaborative shared-care models may further improve accessibility, especially for clients residing in remote or underserved areas. Enhanced communication tools, such as digital case-sharing systems and joint referring veterinarian–oncologist case reviews can also support referring clinicians managing cancer cases locally where travel is impractical. This even has the potential of improving patient outcome, as it has been documented in human medicine that travelling further for care may give worse health outcomes [22]. In particular, the difference travelled between dogs and cats was marked and highlights an important opportunity to implement cat-specific interventions aimed at reducing barriers to referral. Targeted measures to mitigate transport-related stress may meaningfully improve access to specialist oncology care. Practical, low-cost interventions such as structured carrier training, early positive habituation to transport, and the use of pheromone-treated carriers can reduce feline stress during travel and veterinary visits [14,16,38,49]. Additionally, provision of pre-visit anxiolysis where appropriate, and clearer guidance for owners on travel planning, may allow for more equitable oncology service provision between species [11,50].

Overall, this study contributes novel information on travel patterns within UK for specialist veterinary oncology care and underscores the need to consider accessibility when developing future service structures. Further research incorporating broader contextual socioeconomic and demographic data, as well as data on owner-reported motivations, would strengthen understanding and help refine strategies to ensure that the decision to seek specialist cancer care is based on clinical need rather than demographic or geographic constraints.

## Figures and Tables

**Table 1:** Median travel distance (km) and interquartile ranges for animals presenting to a specialist oncology service, stratified by species, breed group, age category, and insurance status. Values represent distance travelled as the crow flies, with median distance, 25th and 75th percentiles, and the interquartile range shown. Numbers in brackets indicate the total number of animals within each subgroup. *p*-values of univariate comparisons performed using the Mann–Whitney U test are presented in the corresponding column. Multivariable *p*-values are derived from the linear regression model using log-transformed distance as the dependent variable. Significant results (*p* < 0.05) are shown in bold.

Variable		Median (km)	25th Percentile (km)	75th Percentile (km)	Interquartile Range (km)	Univariate Analysis (*p*-Value, Mann–Whitney U)	Multivariable Linear Regression Model (*p*-Value)
Species (number)	Dog (2642)	34	12	66	54	**<0.001**	**<0.001**
	Cat (432)	12	8	41	33		
Breed (number)	Pure-breed (2354)	33	7	64	33	**<0.001**	0.589
	Mixed-breed (720)	22	10	60.75	50.75		
Age (number)	<7 years (745)	36	13	67.50	54.5	**0.002**	**0.012**
	≥7 years (2329)	29	11	62	51		
Insurance (number)	Insured (2250)	30	25	60	49	0.078	**0.008**
	Uninsured (824)	33	11	69.75	58.75		

## Data Availability

The datasets presented in this article are not readily available because of UK General Data Protection Regulation (GDPR) legislation and privacy restrictions. The protection of this dataset is also a requirement of the conditions outlined by the Human Ethical Review Committee of the Royal (Dick) School of Veterinary Studies.
